# Missouri

**Published:** 1896-06

**Authors:** 


*Missouri.* The cattle in and adjacent to all large cities in Missouri are to be tested with tuberculin under the direction of the State Board of Health. Dr. T. E. White, State Veterinarian, aided by Dr. Conway, will make the tests.

